# Why acetylcholinesterase inhibitors should be considered disease-modifying drugs for Alzheimer’s disease?

**DOI:** 10.1007/s40520-026-03353-z

**Published:** 2026-03-16

**Authors:** Giovanni Zuliani, Carlo Cervellati, Marco Zuin, Gloria Brombo

**Affiliations:** 1 Department of Translational Medicine and for Romagna, University of Ferrara, Via Luigi Borsari, 46, Ferrara, 44124 Italy; 2 University of Ferrara, Ferrara, Italy

**Keywords:** Alzheimer’s disease, Dementia, Disease-modifying drugs, Acetylcholinesterase inhibitors

## Abstract

Disease-modifying drugs (DMDs) are defined as treatments capable of altering the underlying course of a disease by slowing or modifying its biological progression rather than merely alleviating symptoms. In Alzheimer’s disease (AD), therapeutic options with proven disease-modifying effects remain limited, despite the recent approval of anti-amyloid monoclonal antibodies. Acetylcholinesterase inhibitors (AChEI), currently classified as symptomatic treatments, have accumulated a number of clinical and experimental evidence suggesting a broader role. Long-term clinical and observational studies indicate that AChEI are associated with slower cognitive and functional decline, reduced hippocampal atrophy, lower mortality rates, and improved behavioral and psychological symptoms of dementia. In parallel, preclinical and clinical data show that AChEI may influence multiple key pathogenic mechanisms of AD, including amyloid-β production/aggregation/toxicity, neuroinflammation, glutamatergic excitotoxicity, synaptic dysfunction, and cerebral hypoperfusion. Taken together, these findings support the view that AChEI fulfill the criteria of DMDs, and should be reconsidered as such in the complex therapeutic framework of Alzheimer’s disease.

## Introduction

A disease-modifying drug (DMD) is a drug designed to alter the course or progression of a disease, rather than treating its symptoms, by slowing down, halting or even reversing the underlying biological processes. By modifying the disease process, these drugs are expected to improve long-term outcomes, reduce disability and mortality; this approach is considered a cornerstone in the management of most chronic and progressive illnesses.

Currently, Alzheimer’s disease (AD) has limited options for DMDs; indeed, the only approved DMDs for this frequent type of dementia are monoclonal antibodies, namely aducanumab, lecanemab and donanemab [1]. Aducanumab targets aggregated amyloid-beta (Aβ) plaques, promoting their clearance by microglia, but its development and marketing have recently been stopped; lecanemab binds soluble Aβ protofibrils, aiming to reduce plaque formation and neurotoxicity; donanemab targets a modified form of Aβ (N-terminal pyroglutamate Aβ), facilitating plaque clearance.

However, AD is a highly complex neurodegenerative disorder. Besides Aβ plaque formation (originating from abnormal cleavage of amyloid precursor protein by β- and γ-secretase), AD pathophysiology is multifaceted [2] and involves several interrelated processes including: formation of tau neurofibrillary tangles, synaptic dysfunction and neuronal loss, chronic neuroinflammation (release of pro-inflammatory cytokines by activated microglia/astrocytes), oxidative stress, mitochondrial dysfunction with energy deficits, impaired calcium homeostasis, and vascular alterations, including reduced cerebral blood flow and blood–brain barrier dysfunction. It follows that potential DMDs could act not only at the level of Aβ, but also at many of these pathophysiological processes.

Acetylcholinesterase inhibitors (AChEI) are a primary class of drugs used to manage symptoms in dementia, particularly AD [3]. These inhibitors (namely donepezil, rivastigmine and galantamine) were originally designed to increase cholinergic neurotransmission in the central nervous system (CNS), which is typically reduced in patients with dementia, thereby aiming to improve cognitive function and slow cognitive decline. Over time, clinical and experimental evidence has accumulated both in favour of a possible clinical efficacy of AChEI and of their multiple effects on AD disease processes and mechanisms, particularly for donepezil.

## Clinical effects of AChEI

At least three large meta-analyses have been conducted on randomized controlled trials (RCTs) of AChEI in patients with AD [4–6]. These studies clearly demonstrated that the short-term effect of AChEI is small but statistically significant compared with placebo in terms of cognition, global change and functional outcomes. However, these meta-analyses do not allow a clear understanding of the long-term clinical impact of AChEI on the evolution of AD, since the RCTs had short follow-up periods (generally < 1 year) and most enrolled patients had Mini-Mental State Examination (MMSE) scores well below 24/30, corresponding to moderate to severe AD.

A study by Rountree and colleagues showed that, similarly to RCTs, long-term observational controlled studies indicate that both AChEI monotherapy and combination therapy with memantine are associated with slower cognitive and functional decline and with delayed institutionalization; persistent treatment is associated with slower decline in cognition, daily functioning and global severity, even in patients with advanced disease [7].

Based on data from the National Alzheimer Coordinating Center (NACC), we compared older patients with dementia who were chronically treated or not treated with AChEI [8]. We found that, at the end of follow-up (maximum 13 years), the average decrease in MMSE score was 5.7 points in AD patients treated with AChEI, compared with 10.8 points in those not treated. Subsequently, through an extensive review of the available literature, we showed that AChEI generally slow the rate of cognitive decline in AD [9]. Indeed, AD patients treated with AChEI tend to show a slower deterioration of cognitive scores (16 studies: mean MMSE loss ranging from 0.2 to 1.37 points per year) compared with untreated patients (17 studies: mean MMSE loss ranging from 1.07 to 3.4 points per year) [9].

Beyond cognitive and functional benefits, at least six additional studies (in addition to ours) have reported a significant reduction in total mortality rates in patients with dementia treated with AChEI; this reduction ranged from a minimum of 27% to a maximum of 42% over periods of 2–8 years [8–10], indicating potential broader health benefits of AChEI. Although these data predominantly derive from open or retrospective studies and large health databases, and not from long-term RCTs, they currently represent the best available evidence on the effects of AChEI in real-world settings.

As reviewed by Peres-Gomez Moreta et al., several high-quality reviews provide clear evidence of the effects of AChEI on cognition, global change, behaviour and mortality in patients with AD [11]. Two further clinically relevant aspects supporting the efficacy of AChEI should be highlighted. First, we recently showed that, in patients with AD and severe dementia (MMSE < 10/30) from the NACC Uniform Data Set, treatment with AChEI plus memantine was associated with the best outcomes, including stabilization of cognitive function and reduced mortality rates, suggesting a significant role for AChEI within combination therapy [12]. Second, evidence from two large meta-analyses suggests that AChEI may be useful in the management of behavioural and psychological symptoms of dementia (BPSD) [13, 14]. In this regard, we recently found that, in patients with mild-to-moderate dementia enrolled in the NACC Uniform Data Set, chronic treatment with AChEI (mean follow-up 4.3 years) was associated with a long-term positive effect on BPSD [15]. Specifically, among the Neuropsychiatric Inventory Questionnaire subitems evaluated, hallucinations, agitation/aggression, depression/dysphoria, anxiety, disinhibition and irritability/lability showed significant differences over time in favour of patients treated with AChEI compared with those not treated.

Further indirect evidence supporting the effectiveness of AChEI comes from a Cochrane review conducted by Parsons and colleagues [16]. This large review found that discontinuation of AChEI in patients with AD resulted in worse cognitive, neuropsychiatric and functional outcomes compared with continuation of treatment, although the strength of evidence was generally limited.

Finally, it should be noted that, as reviewed by Ismail et al. in their meta-analysis [17], higher doses of AChEI—particularly donepezil—significantly reduce hippocampal atrophy in patients with AD or mild cognitive impairment, indirectly supporting a potential neuroprotective effect of AChEI.

Overall, AChEI appear to have a “small” but significant clinical effect in the short-, medium- and long-term treatment of patients with mild-to-moderate AD. Most importantly, available data strongly suggest that AChEI slow both clinical progression of AD and hippocampal atrophy and reduce overall mortality. On the other hand, although generally well tolerated, these drugs are not devoid of side effects; there is evidence that AChEI therapy may sometimes affect both physical and psychological health, thus requiring careful clinical monitoring [18].

## Multiple effects of AChEI in the central nervous system

**Fig. 1:**
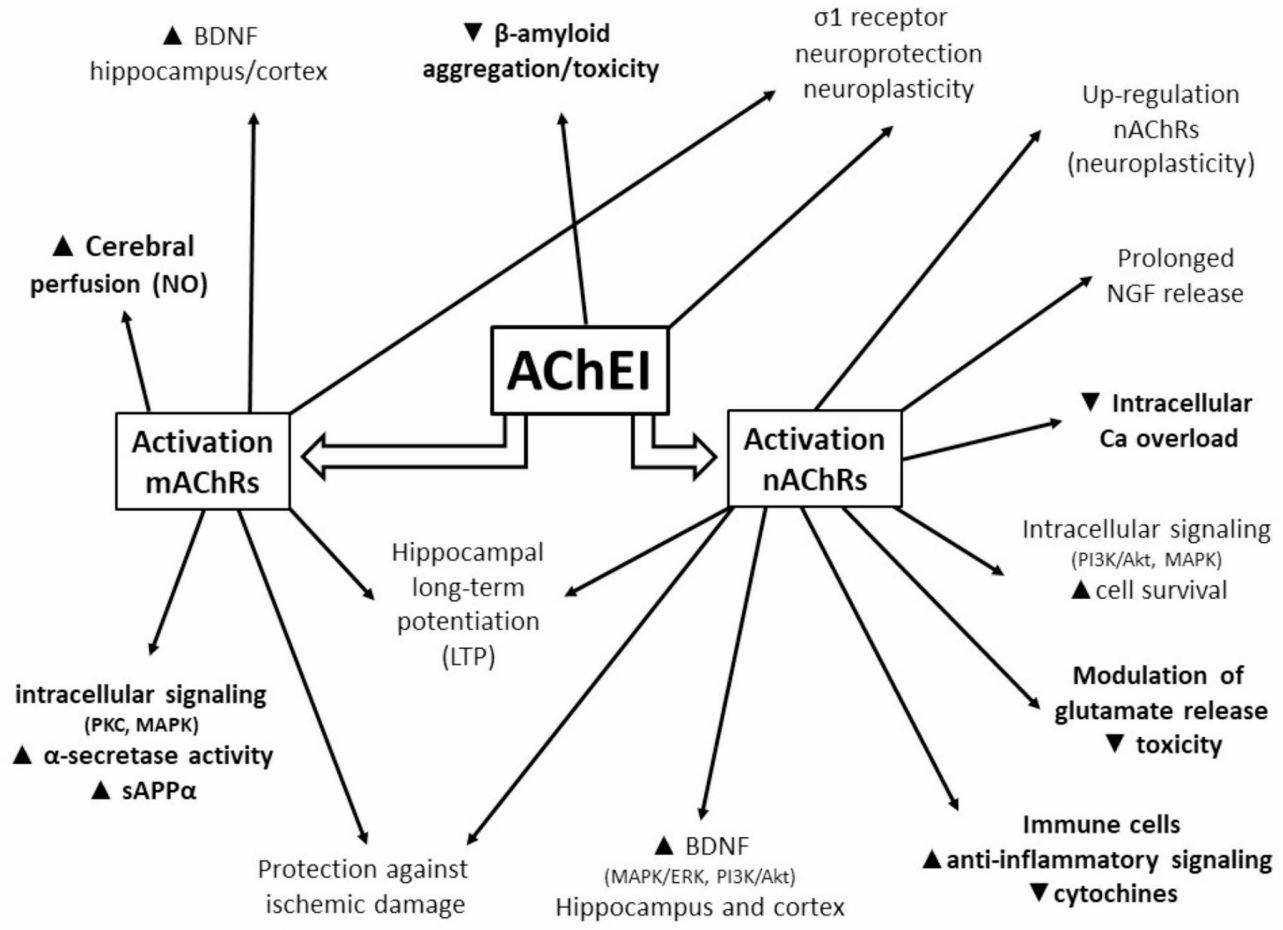
multiple disease-modifying effects of AChEI in Alzheimer’s disease patients

The second point is to verify whether AChEI can interfere with or modify pathological mechanisms directly involved in the complex pathogenesis of AD. Beyond inhibition of acetylcholinesterase (AChE), which increases synaptic acetylcholine (ACh) concentrations and enhances cholinergic signalling, several neuroprotective mechanisms have been demonstrated, mostly in preclinical or early-clinical studies, supporting a potential disease-modifying effect of AChEI (Fig. 1), including [19–41]:

1. Reduction of the neurotoxic effects of β-amyloid. AChE may interact with Aβ peptides, promoting their aggregation into toxic plaques. AChEI may reduce this facilitation of Aβ aggregation, thereby decreasing formation of neurotoxic Aβ fibrils and plaques.
2. Activation of nicotinic acetylcholine receptors (nAChRs). By preventing ACh breakdown, AChEI increase synaptic ACh levels. nAChRs are abundant in the cortex and hippocampus. Chronic stimulation leads to receptor upregulation and enhanced cholinergic signalling, partly mediated by upregulation of nerve growth factor (NGF)-related genes and prolonged NGF release.
3. Modulation of calcium overload. Indirect activation of nAChRs influences intracellular calcium dynamics, helping neurons handle calcium influx and reducing excitotoxic damage.
4. Activation of neuroprotective pathways. nAChR activation stimulates intracellular signalling cascades (e.g. PI3K/Akt and MAPK pathways) that promote cell survival and reduce apoptosis.
5. Reduction of glutamate excitotoxicity. nAChR activation, particularly of the α7 subtype, modulates glutamate release, maintaining balanced neurotransmission and preventing excessive glutamate accumulation.
6. Activation of the cholinergic anti-inflammatory pathway. Increased ACh activates α7 nAChRs on immune cells, inhibiting release of pro-inflammatory cytokines such as TNF-α, IL-1β and IL-6.
7. Interaction with σ1 receptors. Some AChEI, particularly donepezil, bind σ1 receptors, enhancing neuroprotective and neuroplastic effects independent of AChE inhibition.
8. Activation of the non-amyloidogenic APP pathway. AChEI promote muscarinic receptor activation, stimulating α-secretase activity and increasing production of neuroprotective sAPPα.
9. Enhancement of hippocampal long-term potentiation (LTP). AChEI enhance LTP through combined nAChR and mAChR activation, increased glutamate signalling and reduced Aβ toxicity.
10. Increase in BDNF levels. Muscarinic receptor activation upregulates BDNF expression in the hippocampus and cortex.
11. Increase in cerebral blood perfusion. Cholinergic activation of endothelial mAChRs stimulates nitric oxide production, improving cerebral blood flow and neurovascular coupling.
12. Protection against ischemic damage. AChEI enhance neuronal survival pathways, reduce inflammation and oxidative stress, improve perfusion and reduce glutamate-mediated excitotoxicity.

Overall, these data strongly indicate that AChEI can modify several pathological mechanisms activated during the course of AD, both directly and through nAChR and/or mAChR stimulation.

## Discussion

As previously stated [9], the “ideal” drug for the treatment of AD would both stop disease progression and improve cognitive function. Unfortunately, such a drug has not yet been developed, likely because of the complexity of AD pathophysiology and the partial irreversibility of brain damage. Thus, while halting or reversing cognitive decline may be an unrealistic goal, a marked slowing of disease progression represents a more reasonable and clinically relevant target. This is precisely what is expected from monoclonal antibodies developed as DMDs for AD, such as lecanemab and donanemab; however, to date, a clearly clinically meaningful slowing of AD progression has not been convincingly demonstrated, at least within follow-up periods of approximately 1.5 years.

AChEI are currently the most widely prescribed drugs for AD worldwide, with tens of millions of prescriptions. Originating from the cholinergic hypothesis of AD, these drugs were initially developed to increase CNS ACh concentrations and improve cholinergic transmission, and were therefore conceived as symptomatic treatments rather than DMDs. Nevertheless, data from the literature strongly suggest that AChEI exert small but significant clinical effects on cognition, global change and functional outcomes in the short, medium and long term. Moreover, many studies indicate that AChEI slow the clinical progression of mild-to-moderate AD, as well as severe AD when combined with memantine [12], reduce hippocampal atrophy and significantly lower overall mortality rates [8–10]., including dementias, generally aims at two main goals: reducing symptoms and slowing disease progression, thereby delaying disability onset and increasing survival. Available evidence suggests that AChEI may contribute to both of these goals. Importantly, their clinical effects appear comparable in magnitude to those of drugs considered effective in other common chronic degenerative conditions such as chronic obstructive pulmonary disease, chronic heart failure and chronic kidney disease [9].

The key question therefore remains whether AChEI should be considered purely symptomatic drugs or true DMDs. Numerous studies indicate that, despite their modest symptomatic effects, AChEI slow disease progression [7–9] and significantly reduce mortality [8–10]. In addition, substantial preclinical and clinical evidence demonstrates that AChEI exert multiple biological effects on key pathogenic mechanisms of AD. Among these, several mechanisms—highlighted in Fig. 1—represent core aspects of AD pathology, including activation of the non-amyloidogenic APP pathway, reduction of Aβ aggregation and toxicity, modulation of glutamate excitotoxicity and calcium overload, anti-inflammatory signalling mediated by nAChRs, and increased cerebral blood perfusion mediated by mAChRs.

These major mechanisms, together with additional minor effects, may plausibly explain the impact of AChEI on the natural history of AD observed in clinical and epidemiological studies. Although AChEI are currently classified as symptomatic drugs, accumulating evidence suggests that their effects extend well beyond symptom control, encompassing modification of disease progression and interference with pathogenic mechanisms.

From a practical standpoint, redefining AChEI as DMDs may not immediately change clinical practice; however, it could have important implications for prescribing behaviour and long-term treatment adherence, especially considering that many patients with AD never initiate AChEI therapy or discontinue it prematurely.

In conclusion, without overemphasizing the magnitude of their effects, we believe that AChEI should be considered DMDs. Their ability to slow AD progression, reduce overall mortality and act on multiple biological steps of the AD process fulfils the core criteria defining disease-modifying therapies.

## Data Availability

No datasets were generated or analysed during the current study.
